# ghost-tree: creating hybrid-gene phylogenetic trees for diversity analyses

**DOI:** 10.1186/s40168-016-0153-6

**Published:** 2016-02-24

**Authors:** Jennifer Fouquier, Jai Ram Rideout, Evan Bolyen, John Chase, Arron Shiffer, Daniel McDonald, Rob Knight, J Gregory Caporaso, Scott T. Kelley

**Affiliations:** Graduate Program in Bioinformatics and Medical Informatics, San Diego State University, San Diego, CA USA; Center for Microbial Genetics and Genomics, Northern Arizona University, Flagstaff, AZ USA; Department of Biological Sciences, Northern Arizona University, Flagstaff, AZ USA; Institute for Systems Biology, Seattle, WA USA; Department of Pediatrics, and Department of Computer Science and Engineering, University of California San Diego, San Diego, CA USA; Department of Biology, San Diego State University, San Diego, CA USA; San Diego State University, 5500 Campanile Drive, San Diego, CA 92182-4614 USA

## Abstract

**Background:**

Fungi play critical roles in many ecosystems, cause serious diseases in plants and animals, and pose significant threats to human health and structural integrity problems in built environments. While most fungal diversity remains unknown, the development of PCR primers for the internal transcribed spacer (ITS) combined with next-generation sequencing has substantially improved our ability to profile fungal microbial diversity. Although the high sequence variability in the ITS region facilitates more accurate species identification, it also makes multiple sequence alignment and phylogenetic analysis unreliable across evolutionarily distant fungi because the sequences are hard to align accurately. To address this issue, we created *ghost-tree*, a bioinformatics tool that integrates sequence data from two genetic markers into a single phylogenetic tree that can be used for diversity analyses. Our approach starts with a “foundation” phylogeny based on one genetic marker whose sequences can be aligned across organisms spanning divergent taxonomic groups (e.g., fungal families). Then, “extension” phylogenies are built for more closely related organisms (e.g., fungal species or strains) using a second more rapidly evolving genetic marker. These smaller phylogenies are then grafted onto the foundation tree by mapping taxonomic names such that each corresponding foundation-tree tip would branch into its new “extension tree” child.

**Results:**

We applied *ghost-tree* to graft fungal extension phylogenies derived from ITS sequences onto a foundation phylogeny derived from fungal 18S sequences. Our analysis of simulated and real fungal ITS data sets found that phylogenetic distances between fungal communities computed using *ghost-tree* phylogenies explained significantly more variance than non-phylogenetic distances. The phylogenetic metrics also improved our ability to distinguish small differences (effect sizes) between microbial communities, though results were similar to non-phylogenetic methods for larger effect sizes.

**Conclusions:**

The Silva/UNITE-based ghost tree presented here can be easily integrated into existing fungal analysis pipelines to enhance the resolution of fungal community differences and improve understanding of these communities in built environments. The *ghost-tree* software package can also be used to develop phylogenetic trees for other marker gene sets that afford different taxonomic resolution, or for bridging genome trees with amplicon trees.

**Availability:**

*ghost-tree* is pip-installable. All source code, documentation, and test code are available under the BSD license at https://github.com/JTFouquier/ghost-tree.

**Electronic supplementary material:**

The online version of this article (doi:10.1186/s40168-016-0153-6) contains supplementary material, which is available to authorized users.

## Background

While it is now relatively straightforward to profile bacterial diversity in environmental samples using culture-independent approaches [1, 2], protocols for profiling fungal communities are less developed. Fungi play critical roles in many ecosystems. Fungi support plant growth in soils [3], are responsible for enormous agricultural losses [4] can cause serious diseases in humans [5] and can degrade structural integrity of built environments (e.g., homes and office buildings). Fungal spores are also known to cause severe allergic reactions or even toxicity [6]. Despite their importance, fungal diversity remains largely uncategorized.

One issue that has slowed progress in this area is that the small subunit ribosomal RNA (SSU rRNA) gene, the most commonly used marker gene in bacterial community surveys, has evolved relatively slowly in fungi. In practice, this means that reads of fungal SSU rRNA (also commonly referred to as the 18S gene) do not differ enough across taxa to provide a useful level of taxonomic resolution. As a result, there is much interest in using the Internal Transcribed Spacer (ITS) region for profiling fungal communities [7]. Although the ITS is an intergenic sequence, it is still sometimes referred to as a marker gene and we refer to it as a marker gene here for consistency with other projects. The ITS region has a much higher mutation rate (and therefore much more sequence variability across species) than the fungal SSU rRNA, so sequence reads of this region provide much greater taxonomic resolution.

While the higher sequence variability in the ITS marker gene facilitates more accurate taxonomic identification, it also makes multiple sequence alignment of ITS sequences highly unreliable across evolutionarily distant groups of fungi. These unreliable alignments, in turn, result in unreliable phylogenetic trees, which is problematic because phylogenetic information is useful both for taxonomic placement of unknown sequences and for phylogenetic diversity calculations. For example, phylogenetic diversity metrics such as Faith’s phylogenetic diversity (PD) index [8] and UniFrac [9] have improved resolution of community differences relative to their non-phylogenetic analogs that were mostly developed for studying communities of macro-organisms (e.g., Chao1 and Bray-Curtis dissimilarity). The UniFrac distance metric, in particular, has been used to investigate patterns of bacterial diversity in thousands of studies and revealed powerful new insights into the factors driving bacterial community composition [9]. These metrics are very likely useful for studying fungal communities as well, but the lack of phylogenetic resolution in fungal SSU rRNA and the high sequence variability in fungal ITS have prevented their application.

Here, we present *ghost-tree*, an open-source bioinformatics software tool for creating phylogenetic trees using multiple genetic loci. The *ghost-tree* method uses sequences from an evolutionarily conserved marker gene that can be aligned across distant taxonomic lineages to build a “foundation” phylogenetic tree. Then, sequences from a less conserved marker gene that allows for higher taxonomic resolution are aligned within groups of closely related taxa to create “extension” phylogenetic trees that are then grafted onto the foundation tree. The result is the “ghost tree.” In this text, we refer to the software package as *ghost-tree* (italic and hyphenated) and the resulting trees as ghost trees (roman, i.e., not italicized and not hyphenated). The principle behind creating ghost trees is the same as studies using multiple genetic loci to reconstruct phylogenetic relationships (e.g., multilocus sequence typing). However, typical multiple-gene trees require robust sequence alignments of all gene markers across all taxa.

We applied *ghost-tree*, to build foundation trees from aligned databases of fungal 18S rRNA gene sequences and then graft extension trees from fungal ITS to create a single phylogeny that can be used in phylogenetic diversity analyses of fungal communities. Our analysis of simulated and real fungal ITS data sets showed that Principle Coordinates Analysis (PCoA) using ghost tree-based UniFrac distances explained substantially more of the variance in the data than non-phylogenetic distances. The phylogenetic approach also significantly improved the ability to distinguish small effect sizes compared with non-phylogenetic metrics using ANOSIM-based group comparisons, though non-phylogenetic methods achieved slightly higher R values for detecting a large effect. Our hybrid 18S/ITS fungal ghost tree and the *ghost-tree* software package that can be used to develop phylogenetic trees for other sets of marker genes can be downloaded from GitHub at: https://github.com/JTFouquier/ghost-tree.

## Implementation

### *ghost-tree* workflow

**Fig. 1:**
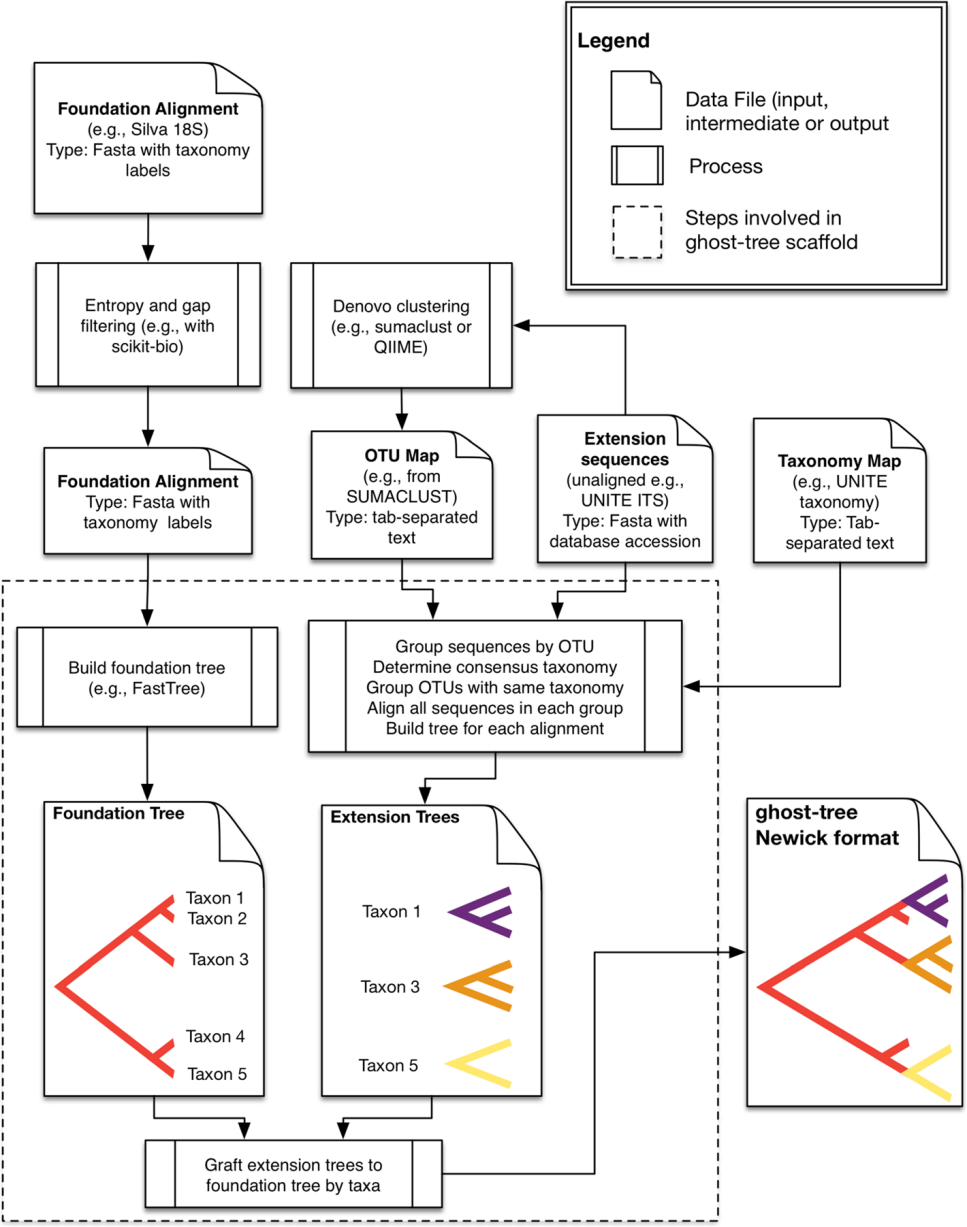
*ghost-tree* workflow diagram

*ghost-tree* takes as input (1) the *Foundation Alignment* (for example, the Silva 18S alignment) where sequences are annotated with taxonomy; (2) the *Extension Sequence Collection* (for example, unaligned ITS sequences from the UNITE database); and (3) a *taxonomy map*, which contains taxonomic annotations of the sequences in (2). The Foundation Alignment is filtered by *ghost-tree* using scikit-bio (scikit-bio.org) to remove highly gapped and high entropy positions. Next, FastTree [10] is used, with the Jukes and Cantor model of DNA evolution [11], to build a phylogenetic tree from the resulting filtered alignment. This is the *Foundation Tree*. In parallel, the *Extension Sequence Collection* can be clustered with SUMACLUST, an open source OTU clustering software package (https://git.metabarcoding.org/obitools/sumaclust/wikis/home/), resulting in an *operational taxonomic unit (OTU) map* that groups sequences into OTUs by percent identity. For each *Extension Sequence OTU*, a consensus taxonomy is determined from the Taxonomy Map, and OTUs with the same consensus taxonomy are combined into a single OTU. The sequences in each OTU are then aligned using MUSCLE with diagonal optimization of the first iteration and two maximum iterations, which is suitable for closely related sequences [12]. FastTree is then applied to these alignments to build an *Extension Tree* for each alignment. This process of “alignment and tree building” is applied to all OTUs in the *Extension Sequence Collection*. Each OTUs’ consensus taxonomy is associated with the root of the *Extension Tree.* The taxa at the root of the extension trees are then used to graft the *Extension Tree* onto the tip in the *Foundation Tree* with the same taxonomy, resulting in the ghost tree (see illustrations in Fig. 1). We applied *ghost-tree* to build a phylogenetic tree from Silva (Ver. SSU 119.1) 18S sequences (our foundation) [13] and UNITE (Ver. 12_11_otus) ITS sequences (our extensions) [14]. This tree is available in the *ghost-tree* GitHub repository. This workflow is illustrated in Fig. 1.

### Test data set

To compare diversity analysis results, we began with two ITS profile data sets: one containing 20 human saliva samples [15] and one containing 16 public restroom floor samples [16]. Both sample sets used the ITS-1F forward primer (5′-CTTGGTCATTTAGAGGAAGTAA-3′) sequence [17] and the ITS2 reverse primer (5′-GCTGCGTTCTTCATCGATGC-3′) sequences [18] to generate ITS1 sequence reads. All analyses referencing Python scripts described below were performed in MacQIIME 1.9.0-20140227 ([19]; http://www.wernerlab.org/software/macqiime). The public restroom floor sequences were generated on an Ilumina MiSeq and the saliva on a GS-FLX pyrosequencer. Sequence data was combined for both studies after demultiplexing (split_libraries.py was performed by the authors on the public restroom floor sequences; already demultiplexed data was obtained from NCBI SRA for the saliva data). OTUs were picked using uclust-based closed-reference OTU picking (pick_otus.py) with UNITE OTUs v12_11 as the reference database and the most abundant sequence in each OTU was selected as the OTU representative sequence (pick_rep_set.py).

The BIOM table was then filtered using filter_otus_from_otu_table.py to contain only OTUs with accession numbers present in the ghost tree. To create a data set with a known small effect size, we used simsam.py, which creates a specified number of phylogenetically similar simulated samples using a phylogenetic tree and the filtered BIOM table. Specifically, the small effect we looked for was whether simulated samples derived from a single source sample were more similar to each other than to simulated communities derived from different source samples. Ten simulated samples were created for each of the 36 source samples with 0.6 dissimilarity (d) using simsam.py, resulting in our “simulated BIOM table,” and a simulated mapping file with metadata for all 360 resulting samples. Simulated BIOM tables were created using *ghost-tree* (which we refer to as the *ghost-tree-*Simulated Communities, or GTSCs) and repeated using FastTree with ITS sequences aligned with MUSCLE (which we refer to as the FastTree-Simulated Communities, or FTSCs).

### Principal coordinates analyses

PCoA plots for unweighted and weighted UniFrac were created using beta_diversity_through_plots.py with the appropriate simulated mapping file, simulated BIOM table, and ghost tree or our FastTree as the reference tree. PCoA plots for the Jaccard distance [20], a qualitative non-phylogenetic diversity metric, and Bray-Curtis distance [21], a quantitative, non-phylogenetic diversity metric, were created separately by using three scripts. First, beta_diversity.py was run with methods “binary_jaccard” and “bray_curtis”, respectively, on the simulated BIOM table to produce two distance matrices (DMs). Next, principal_coordinates.py was applied to those DMs to produce principal coordinates (PC) matrices. Finally, make_emperor.py was run using the PC files and the simulated mapping file to produce PCoA plots for Jaccard and Bray-Curtis. This process was repeated for unsimulated samples and for both FTSCs and GTSCs.

### Diversity calculations and statistics

To test whether simulated samples derived from the same source sample were more similar than those derived from different source samples (the small effect), per-environment OTU tables (saliva and restroom floor) were created using split_otu_table.py. ANOSIM was computed using compare_categories.py to compare the distribution of distances between samples with the same source to the distribution of samples with a different source. Six distance matrix calculations were created for both FTSCs and GTSCs using the appropriate simulated mapping file with 999 permutations: Jaccard, Bray-Curtis, unweighted UniFrac with FastTree, weighted UniFrac with FastTree, unweighted UniFrac with *ghost-tree*, and weighted UniFrac with *ghost-tree*. The Jaccard distance is a qualitative non-phylogenetic diversity metric where no tree is required. The Bray-Curtis distance is a quantitative, non-phylogenetic diversity metric where no tree is required. The weighted UniFrac metric includes information on the abundance of various taxa in addition to the phylogenetic tree, while the unweighted UniFrac only includes the phylogenetic information (for details see [9]). The unweighted and weighted FastTree distances are calculated with a phylogenetic tree based on a FastTree phylogenetic analysis of only the ITS data aligned using MUSCLE.

To test whether samples could be differentiated by their environment type (*restroom* or *saliva*, the large effect), ANOSIM was computed using compare_categories.py on each of the six distance matrices for the simulated and real BIOM tables using the appropriate (either simulated or unsimulated) mapping file, with 999 permutations. This analysis was performed for simulated and unsimulated sample sets.

### *ghost-tree* software

*ghost-tree* is hosted on GitHub under the BSD open source software license. It is implemented in Python, using scikit-bio (www.scikit-bio.org) and Click (http://click.pocoo.org/), and adheres to the PEP8 Python style guide. *ghost-tree* is subject to continuous integration testing using Travis CI which, on each pull request, runs unit tests with nose, monitors code style using flake8, and monitors test coverage with coveralls.

## Results and discussion

To evaluate whether *ghost-tree* supports improved sample resolution in studies of fungal community analysis, we evaluated its ability to detect small and large effect sizes. We analyzed two real-world (referred to in the tables and figures as unsimulated/real) ITS sequence data sets: one a collection of human saliva samples and one of surfaces in public restrooms. The communities in human saliva and on restroom surfaces were expected to differ substantially from one another, and we further expected that any of the metrics should be able to detect these differences. This was, therefore, considered the “large effect size” data set.

To generate data sets with known small effect sizes, we simulated 10 samples for each saliva and restroom sample (the source samples), using QIIME’s simsam.py workflow. The simsam.py workflow generates sample replicates that are phylogenetically similar to their source sample. Metrics that can differentiate closely related samples should assign smaller distances between samples that are simulated from the same source sample and larger distances between samples that are simulated from different source samples. We considered the grouping of each set of simulated samples that were derived from the same source to be the “small effect size” data set. Because simsam.py requires a phylogenetic tree to simulate phylogenetically similar samples, we have simulated communities using trees generated both with *ghost-tree* and FastTree and evaluated each resulting set of samples with both the *ghost-tree-*generated phylogeny and the FastTree-generated phylogeny. Because of the limitations with fungal community analysis noted above, it is difficult to obtain data sets with known large and small effect sizes that also sequence the same region of the ITS, so we used this approach to generate both small and large effect size samples.

### Large effect size analyses

**Fig. 2:**
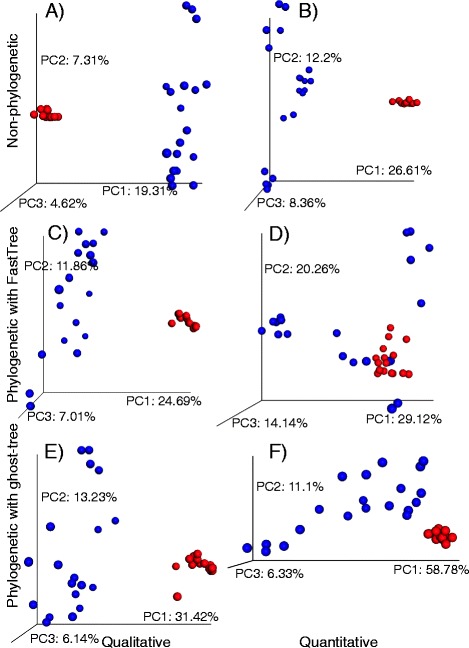
Principal coordinates comparing *unsimulated* (real) samples based on **a** Jaccard distances, **b** Bray-Curtis distances, **c** unweighted UniFrac distances where trees are computed using FastTree, **d** weighted UniFrac distances where trees are computed using FastTree, **e** unweighted UniFrac distances where trees are computed using *ghost-tree*, and **f** weighted UniFrac distances where trees are computed using *ghost-tree. Blue points* are simulated and real human saliva samples, and *red points* are simulated and real restroom surface samples. Plots were made using EMPeror software [26]

Analysis of the unsimulated (real) data with large effect sizes found that, as expected, both non-phylogenetic and phylogenetic methods readily distinguished fungal communities determined from human saliva and restroom floors (Table 1; Fig. 2). However, while the non-phylogenetic methods had the highest *R* values, they explained less of the variation than the phylogenetic metrics computed with *ghost-tree* or FastTree phylogenies. The phylogenetic metrics computed with *ghost-tree’s* tree also explained slightly more of the variation (Table 1, unweighted UniFrac percent explained = 50.790; weighted UniFrac percent explained = 76.210; Fig. 2e, f) than the phylogenetic metrics based on the FastTree phylogeny (Table 1, unweighted UniFrac percent explained = 43.56; weighted UniFrac percent explained = 63.52; Fig. 2c, d). Weighted UniFrac with FastTree also proved less able to distinguish large effect sizes (Table 1, ANOSIM *R* = 0.263; Fig. 2d) than the weighted UniFrac with *ghost-tree* (Table 1, ANOSIM *R* = 0.463; Fig. 2f). The same was true for unweighted UniFrac, though the difference in *R* value was negligible.

**Table 1:** Quantitative group comparisons using ANOSIM and PCoA to analyze large effect sizes (between environments) of simulated and unsimulated human saliva and public restroom floor samples

	Test statistic (*R*)	*p* value	% explained
Unsimulated (real) data community analysis			
Jaccard (Fig. 2a)	0.865	0.001	31.24
Bray-Curtis (Fig. 2b)	0.849	0.001	47.17
Unweighted UniFrac with FastTree (Fig. 2c)	0.734	0.001	43.56
Weighted UniFrac with FastTree (Fig. 2d)	0.263	0.001	63.52
Unweighted UniFrac with *ghost-tree* (Fig. 2e; Additional file 1: Figure S1A)	0.753	0.001	50.79
Weighted UniFrac with *ghost-tree* (Fig. 2f; Additional file 1: Figure S1B)	0.463	0.001	76.21
Unweighted UniFrac with *ghost-tree* 0 branch foundation (Additional file 1: Figure S1C)	0.730	0.001	50.61
Weighted UniFrac with *ghost-tree* 0 branch foundation (Additional file 1: Figure S1D)	0.458	0.001	76.91
Unweighted UniFrac with *ghost-tree* 0 branch extensions (Additional file 1: Figure S1E)	0.700	0.001	66.11
Weighted UniFrac with *ghost-tree* 0 branch extensions (Additional file 1: Figure S1F)	0.453	0.001	67.97
Simulated data community analysis			
Jaccard to analyze FTSCs (Fig. 3a)	0.191	0.001	3.05
Bray-Curtis to analyze FTSCs (Fig. 3b)	0.191	0.001	2.71
Jaccard to analyze GTSCs (Fig. 3c)	0.036	0.001	1.63
Bray-Curtis to analyze GTSCs (Fig. 3d)	0.036	0.001	2.43
Unweighted UniFrac with FastTree to analyze FTSCs (Fig. 3e)	0.675	0.001	22.10
Weighted UniFrac with FastTree to analyze FTSCs (Fig. 3f)	0.255	0.001	43.69
Unweighted UniFrac with FastTree to analyze GTSCs (Fig. 3g)	0.298	0.001	68.87
Weighted UniFrac with FastTree to analyze GTSCs (Fig. 3h)	0.150	0.001	54.08
Unweighted UniFrac with *ghost-tree* to analyze FTSCs (Fig. 3i)	0.302	0.001	27.72
Weighted UniFrac with *ghost-tree* to analyze FTSCs (Fig. 3j)	0.117	0.001	35.55
Unweighted UniFrac with *ghost-tree* to analyze GTSCs (Fig. 3k)	0.580	0.001	20.40
Weighted UniFrac with *ghost-tree* to analyze GTSCs (Fig. 3l)	0.307	0.001	44.98

Note: For unsimulated samples, sample size is 36, and two groups were analyzed using 999 permutations. For simulated samples, sample size is 360, and two groups were analyzed using 999 permutations. The test statistic (*R*), *p* value, and percent variation explained in the first the PCoA axes are presented for each comparison

**Fig. 3:**
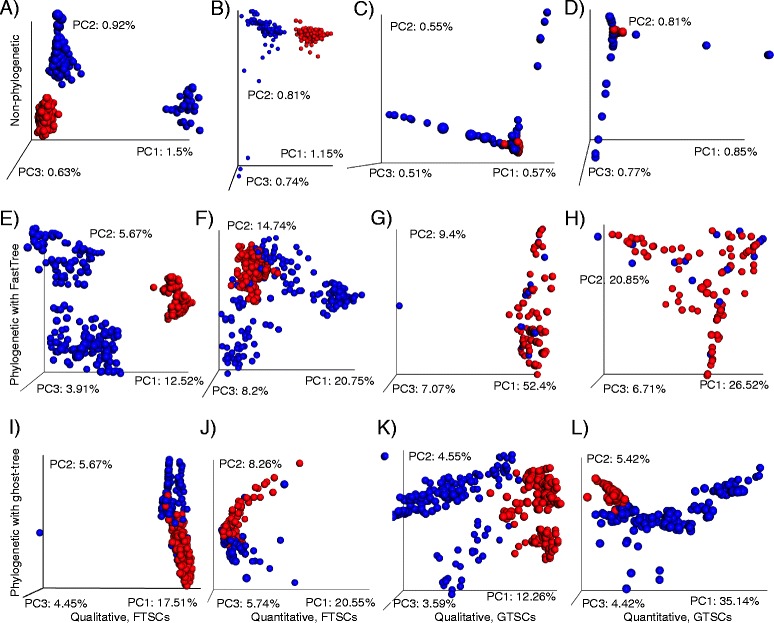
Principal coordinates comparing *simulated* samples based on **a** Jaccard distances to analyze FastTree-simulated communities (FTSCs), **b** Bray-Curtis distances to analyze FTSCs, **c** Jaccard distances to analyze *ghost-tree*-simulated communities (GTSCs), **d** Bray-Curtis distances to analyze GTSCs, **e** unweighted UniFrac distances where trees are computed using FastTree to analyze FTSCs, **f** weighted UniFrac distances where trees are computed using FastTree to analyze FTSCs, **g** unweighted UniFrac distances where trees are computed using FastTree to analyze GTSCs, **h** weighted UniFrac distances where trees are computed using FastTree to analyze GTSCs, **i** unweighted UniFrac distances where trees are computed using *ghost-tree* to analyze FTSCs, **j** weighted UniFrac distances where trees are computed using *ghost-tree* to analyze FTSCs, **k** unweighted UniFrac distances where trees are computed using *ghost-tree* to analyze GTSCs, and **l** weighted UniFrac distances where trees are computed using *ghost-tree* to analyze GTSCs. *Blue points* are simulated and real human saliva samples, and *red points* are simulated and real restroom surface samples. Plots were made using EMPeror software [26]

The analysis of the FTSC- and GTSC-simulated data with large effect sizes found a similar pattern. All methods were able to differentiate between salivary and restroom floor communities, though the non-phylogenetic methods had lower *R* values and explained only a small portion of the overall variation (Table 1; Fig. 3a–d). The unweighted and weighted UniFrac distances based on FastTree and *ghost-tree* performed much better on the FTSC and GTSC communities (Table 1; Fig. 3e–l). Unsurprisingly, the results were the strongest when the phylogenetic distances were computed for a simulated data using the same phylogeny that was used to simulate it. For instance, unweighted UniFrac using the ghost tree had a higher ANOSIM *R* value and explained more of the overall variation using the GTSC (Table 1; Fig. 3k) than the FTSC (Table 1; Fig. 3i). It is therefore important to consider how each metric performed with when a different tree was used for simulating the community and computing phylogenetic distances. Interestingly, while the UniFrac metrics based on the FastTree phylogeny had the best statistics, they showed no clear separation of the communities not only in the PCoA plots, particularly when using the GTSCs (Fig. 3g, h), but also in the weighted analysis using the FTSCs (Fig. 3f). The reason for this discrepancy is unknown, but the visual separation is clearly much better using the ghosttree with both the FSTCs and GTSCs.

### Small effect size analyses

Table 2 details the result of ANOSIM analysis comparing simulated (small effect size) data within each of the two sample types. The results show that the phylogenetic metrics are better at distinguishing samples with very small effect size differences compared with the non-phylogenetic metrics. While the *p* values were highly significant for all the tests (except FastTree phylogeny with GTSCs), the ANOSIM *R* values were considerably higher using the unweighted and weighted UniFrac with both *ghost-tree* and FastTree. Interestingly, the UniFrac analysis using FastTree on data simulated using the FastTree phylogeny (FTSC) had the highest *R* value of all the tests for both the restroom- and saliva-simulated datasets (Table 2). However, when the UniFrac distances using FastTree were generated for the GTSCs, the *R* values dropped considerably and became non-significant in two of the tests (unweighted and weighted UniFrac using FastTree to analyze GTSCs; Table 2). On the other hand, the unweighted and weighted UniFrac *ghost-tree* metrics appeared to be much more robust regardless of the underlying phylogeny used to create the simulated communities.

**Table 2:** Quantitative group comparisons using ANOSIM to analyze small effect sizes (within environments) of simulated human saliva and public restroom floor samples

	Test statistic (*R*)	*p* value
Restroom samples		
Non-phylogenetic methods		
Jaccard to analyze FTSCs	0.225	0.001
Bray-Curtis to analyze FTSCs	0.239	0.001
Jaccard to analyze GTSCs	0.053	0.001
Bray-Curtis to analyze GTSCs	0.056	0.001
FastTree		
Unweighted UniFrac with FastTree to analyze FTSCs	0.673	0.001
Weighted UniFrac with FastTree to analyze FTSCs	0.798	0.001
Unweighted UniFrac with FastTree to analyze GTSCs	0.038	0.057
Weighted UniFrac with FastTree to analyze GTSCs	-0.001	0.518
* ghost-tree*		
Unweighted UniFrac with *ghost-tree* to analyze FTSCs	0.125	0.001
Weighted UniFrac with *ghost-tree* to analyze FTSCs	0.073	0.001
Unweighted UniFrac with *ghost-tree* to analyze GTSCs	0.619	0.001
Weighted UniFrac with *ghost-tree* to analyze GTSCs	0.655	0.001
Saliva samples		
Non-phylogenetic methods		
Jaccard to analyze FTSCs	0.250	0.001
Bray-Curtis to analyze FTSCs	0.253	0.001
Jaccard to analyze GTSCs	0.032	0.001
Bray-Curtis to analyze GTSCs	0.032	0.001
FastTree		
Unweighted UniFrac with FastTree to analyze FTSCs	0.852	0.001
Weighted UniFrac with FastTree to analyze FTSCs	0.756	0.001
Unweighted UniFrac with FastTree to analyze GTSCs	0.031	0.001
Weighted UniFrac with FastTree to analyze GTSCs	0.023	0.001
* ghost-tree*		
Unweighted UniFrac with *ghost-tree* to analyze FTSCs	0.125	0.001
Weighted UniFrac with *ghost-tree* to analyze FTSCs	0.068	0.001
Unweighted UniFrac with *ghost-tree* to analyze GTSCs	0.524	0.001
Weighted UniFrac with *ghost-tree* to analyze GTSCs	0.596	0.001

Note: For restroom sample diversity metrics, sample size is 160, and 16 groups were analyzed using 999 permutations. For saliva sample diversity metrics, sample size is 200, and 20 groups were analyzed using 999 permutations

Finally, to determine the relative influence of the phylogenetic information from the foundation tree (18S phylogeny) and the extension trees (ITS phylogenies), we tested how the removal of all branch lengths in each (and therefore the phylogenetic information contributed by each) affected our ability to detect our small effect sizes. Additional file 1: Figure S1 shows the PCoA visualizations of these analyses. Removal of both the foundation phylogenetic information and the extension tree information lowered the resulted ANOSIM *R* values in both unweighted and weighted UniFrac analyses (Table 1) suggesting that both contributed to the analysis. However, the total variation explained dropped the most when the extension tree phylogenetic information was removed indicating that, at least for these data, the ITS extension trees contributed the most to the analysis with these particular environments.

## Conclusions

Fungi play an integral role in many ecosystems and have known impacts on building materials, agricultural soils, and human health. Here, we show how the *ghost-tree* approach allows incorporation of phylogenetic information into fungal community analysis based on the ITS marker sequence. Phylogenetic-based analysis (e.g., UniFrac) of bacterial communities has proven a powerful means for detecting associations between environmental conditions and microbial diversity, assessing the temporal dynamics of communities, and detecting shifts in microbial community structure after experimental treatments [22–24]. Our results show that incorporating phylogeny into diversity metrics can enhance the resolution at which we can detect differences among fungal communities and should, therefore, improve our understanding of fungal ecosystems in the built environment and other settings.

Phylogenetic analyses based on single marker genes should always be used cautiously, but, as with the analysis of bacterial communities, the inclusion of phylogenetic information seems to improve fungal community diversity analysis. One limitation to our approach is that branch lengths are not scaled when grafting extension trees onto the foundation tree, which would control for different rates of evolution across the two marker genes being combined. We next plan to explore how to best scale branch lengths to further improve diversity analysis, and this will be added in a future iteration of *ghost-tree*.

The Silva/UNITE-based ghost tree developed for the analyses presented here is publicly accessible and can be easily integrated into a user’s existing fungal analysis pipeline. The *ghost-tree* software package can also be used to develop phylogenetic trees for other marker gene sets that provide different taxonomic resolution or for bridging genome trees with amplicon trees. Marker gene sequence databases such as SILVA and UNITE provide reference sequences and taxonomy that are widely used for fungal community analysis. For other marker genes where reference trees are not widely distributed, or when researchers wish to integrate sequences that are not represented in a reference database as with open-reference OTU picking workflows [25], trees must be constructed from the marker sequence reads if phylogenetic analyses will be performed. The ghost tree method facilitates development of phylogenetic trees that can be distributed with reference sequences or built from marker sequence reads in conjunction with a reference database.

## Availability and requirements

All source code, documentation (including installation requirements) and test code are available under the BSD license at https://github.com/JTFouquier/ghost-tree.

## Additional file

Additional file 1: Figure S1. Principal Coordinates comparing *unsimulated* (real) samples based on (a) unweighted UniFrac distances where trees are computed using *ghost-tree*, (b) weighted UniFrac distances where trees are computed using *ghost-tree*, (c) unweighted UniFrac distances where trees are computed using *ghost-tree*, 0-branch length-foundation, (d) weighted UniFrac distances where trees are computed using *ghost-tree*, 0-branch-length foundation, (e) unweighted UniFrac distances where trees are computed using *ghost-tree*, 0-branch-length extensions, (f) weighted UniFrac distances where trees are computed using *ghost-tree*, 0-branch-length extensions. Blue points are simulated and real human saliva samples, and red points are simulated and real restroom surface samples. Plots were made using EMPeror software [25]. (PDF 522 kb)

## References

[1] Human Microbiome Project Consoritum. A framework for human microbiome research. Nature. 2012;486:215–21. 10.1038/nature11209 PMC3377744 22699610

[2] Su C, Lei L, Duan Y, Zhang K-Q, Yang J (2012). Culture-independent methods for studying environmental microorganisms: methods, application, and perspective. Applied Microbial Biotechnol.

[3] van der Heijden MGA, Klironomos JN, Ursic M, Moutoglis P, Streitwolf-Engel R, Boller T (1998). Mycorrhizal fungal diversity determines plant biodiversity, ecosystem variability and productivity. Nature.

[4] Johansson JF, Paul LR (2004). Microbial interactions in the mycorrhizosphere and their significance for sustainable agriculture. FEMS Microbiol Ecol.

[5] Centers for Disease Control and Prevention (2009). CDC and fungal diseases: why are fungal diseases a public health issue?.

[6] Crook B, Burton N (2010). Indoor moulds, sick building syndrome and building related illness. Fungal Biol Rev.

[7] Schoch, Seifert, Huhndorf, et al. Nuclear ribosomal internal transcribed spacer (ITS) region as a universal DNA barcode marker for Fungi. Proc. Natl. Acad. Sci. USA. 2012, 109:6241–6. 10.1073/pnas.1117018109 PMC3341068 22454494

[8] Faith DP (1992). Conservation evaluation and phylogenetic diversity. Biol Conserv.

[9] Lozupone C, Knight R (2005). UniFrac: a new phylogenetic method for comparing microbial communities. Appl Environ Microbiol.

[10] Price MN, Dehal PS, Arkin AP (2009). FastTree: computing large minimum evolution trees with profiles instead of a distance matrix. Mol Biol Evol.

[11] Jukes TH, Cantor CR. Evolution of protein molecules. Mamm Protein Metab. 1969, 21-132.

[12] Edgar RC (2004). MUSCLE: multiple sequence alignment with high accuracy and high throughput. Nucleic Acids Res.

[13] Pruesse E, Quast C, Knittel K, Fuchs B, Ludwig W, Peplies J (2007). SILVA: a comprehensive online resource for quality checked and aligned ribosomal RNA sequence data compatible with ARB. Nucleic Acids Res.

[14] Kõljalg U, Nilsson R, Abarenkov K, Tedersoo L, Taylor A, Bahram M (2013). Towards a unified paradigm for sequence-based identification of fungi. Mol Ecol.

[15] Ghannoum M, Jurevic R, Mukherjee P, Cui F, Sikaroodi M, Naqvi A, et al. Characterization of the oral fungal microbiome (mycobiome) in healthy individuals. PLoS Pathog. 2010;6:e1000713. 10.1371/journal.ppat.1000713 PMC2795202 20072605

[16] Fouquier J, Schwartz T (2016). Kelley ST.

[17] Gardes M, Bruns TD. ITS primers with enhanced specificity for basidiomycetes—application to the identification of mycorrhizae and rusts. Mol Ecol. 1993;2(2):113–8. 10.1111/j.1365-294x.1993.tb00005.x 8180733

[18] White TJ, Bruns T, Lee S, Taylor JW. Amplification and direct sequencing of fungal ribosomal RNA genes for phylogenetics. PCR Protoc. 1990, 315-322.

[19] Caporaso G, Kuczynski J, Stombaugh J, et al. QIIME allows analysis of high-throughput community sequencing data. Nat Methods. 2010;7:335–6. 10.1038/nmeth.f.303 PMC3156573 20383131

[20] Jaccard P. The distribution of the flora in the alpine zone. New Phytol. 1912;11(2):37-50.

[21] Bray JR, Curtis JT. An ordination of upland forest communities of southern Wisconsin. Ecol Monogr. 1957;27:325–49.

[22] Caporaso JG, Lauber CL, Costello EK. Moving pictures of the human microbiome. Genome Biol. 2011;12(5):R50 10.1186/gb-2011-12-5-r50 PMC3271711 21624126

[23] Gibbons SM, Schwartz T, Fouquier J. Ecological succession and viability of human-associated microbiota on restroom surfaces. Appl Environ Microbiol. 2015;81(2):765–73. 10.1128/AEM.03117-14 PMC4277569 25398865

[24] Lauber CL, Hamady M, Knight R, Fierer N. Pyrosequencing-based assessment of soil pH as a predictor of soil bacterial community structure at the continental scale. Appl Environ Microbiol. 2009;75:5111–20. 10.1128/AEM.00335-09 PMC2725504 19502440

[25] Rideout JR, He Y, Navas-Molina JA, Walters WA. Subsampled open-reference clustering creates consistent, comprehensive OTU definitions and scales to billions of sequences. PeerJ. 2014;2:e545. 10.7717/peerj.545 PMC4145071 25177538

[26] Vázquez-Baeza Y, Pirrung M, Gonzalez A, Knight R. A tool for visualizing high-throughput microbial community data. Giga Sci. 2013;2:16. 10.1186/2047-217X-2-16 PMC4076506 24280061

