# Supplementary figures and images for: ghost-tree: creating hybrid-gene phylogenetic trees for diversity analyses
Source: Microbiome. 2016 Feb 24;4:11. doi: 10.1186/s40168-016-0153-6 (PMC4765138; doi:10.1186/s40168-016-0153-6)

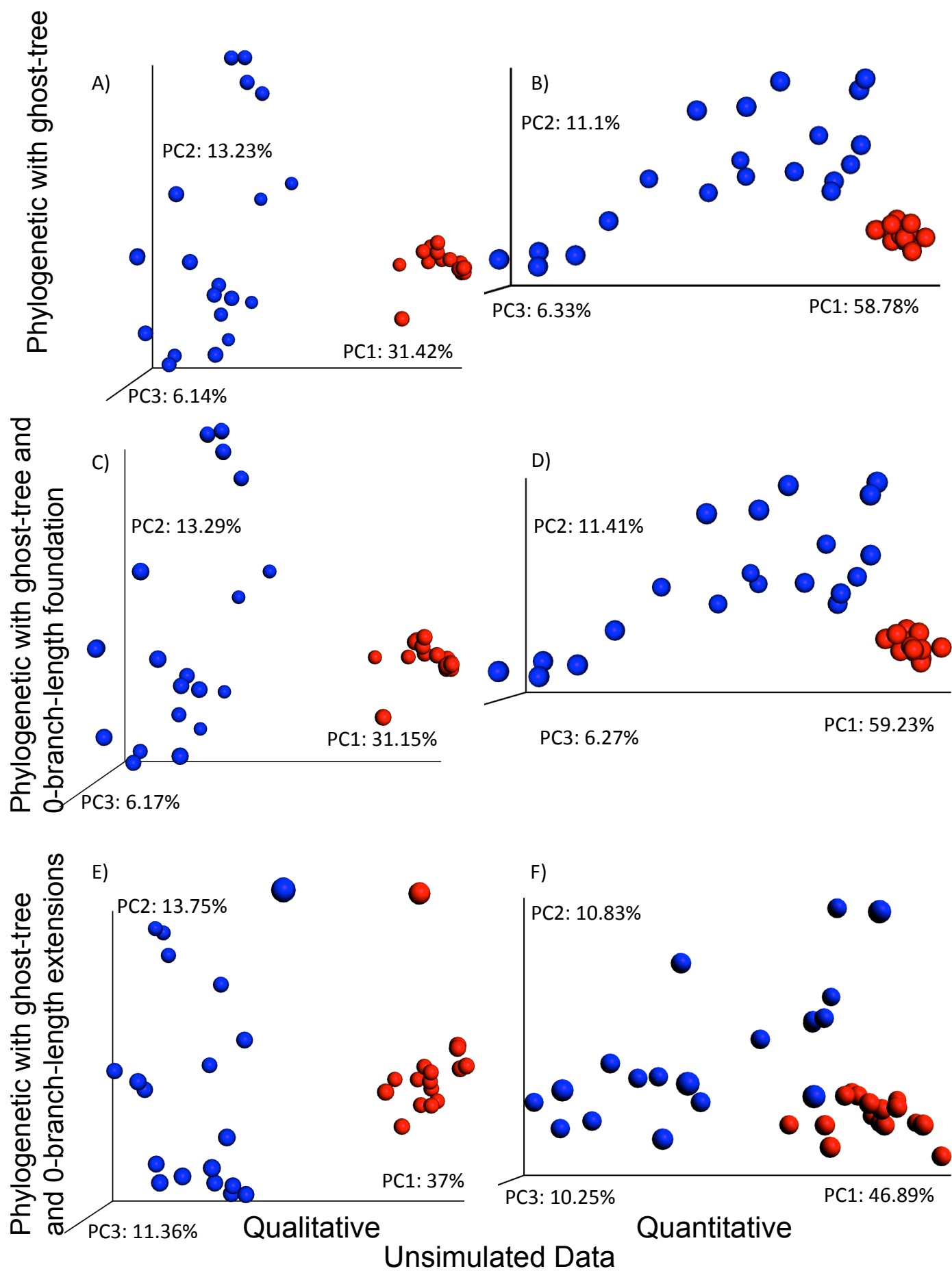

Supplement: Additional file 1: Figure S1. — Principal Coordinates comparing unsimulated (real) samples based on (a) unweighted UniFrac distances where trees are computed using ghost-tree, (b) weighted UniFrac distances where trees are computed using ghost-tree, (c) unweighted UniFrac distances where trees are computed using ghost-tree, 0-branch length-foundation, (d) weighted UniFrac distances where trees are computed using ghost-tree, 0-branch-length foundation, (e) unweighted UniFrac distances where trees are computed using ghost-tree, 0-branch-length extensions, (f) weighted UniFrac distances where trees are computed using ghost-tree, 0-branch-length extensions. Blue points are simulated and real human saliva samples, and red points are simulated and real restroom surface samples. Plots were made using EMPeror software [25]. (PDF 522 kb) [file 40168_2016_153_MOESM1_ESM.pdf]
